# Supplementary material for: Dysfunctional Chondroitin 4-O-Sulfotransferase-1 Impairs Cellular Redox State and Promotes Tau Aggregation
Source: Cells. 2025 Oct 28;14(21):1686. doi: 10.3390/cells14211686 (PMC12607407; doi:10.3390/cells14211686)

Supplementary Figure S1

Fig. 2(b)

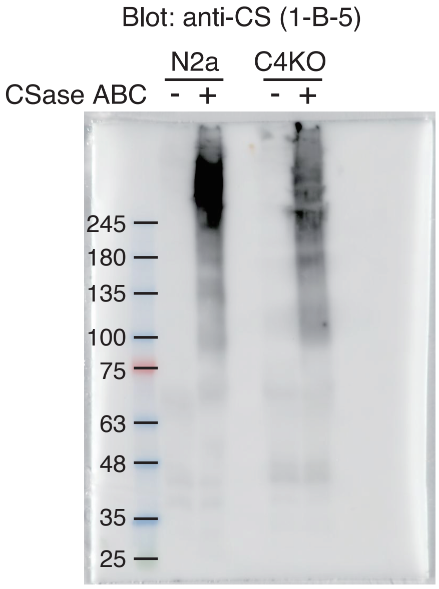

Fig. 2(c)

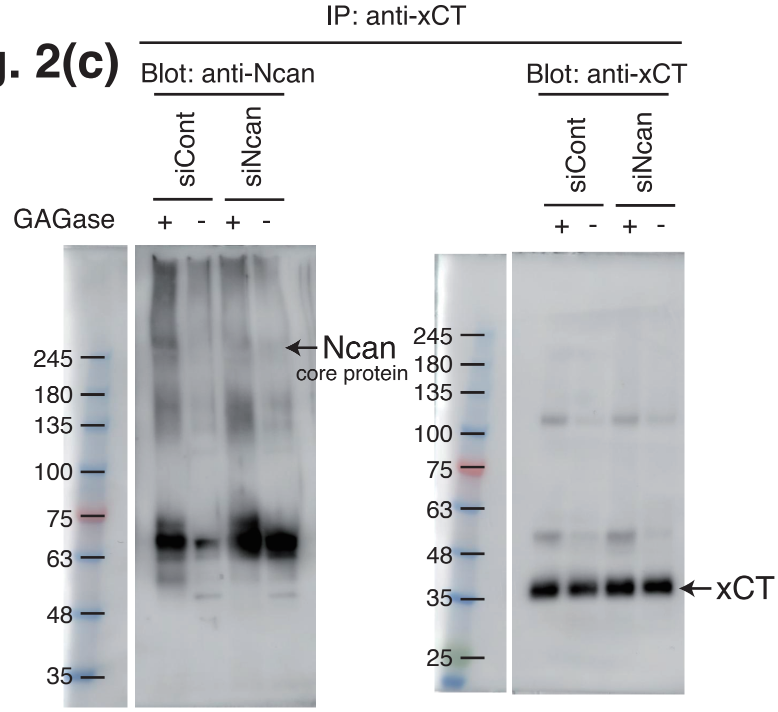

Fig. 2(d)

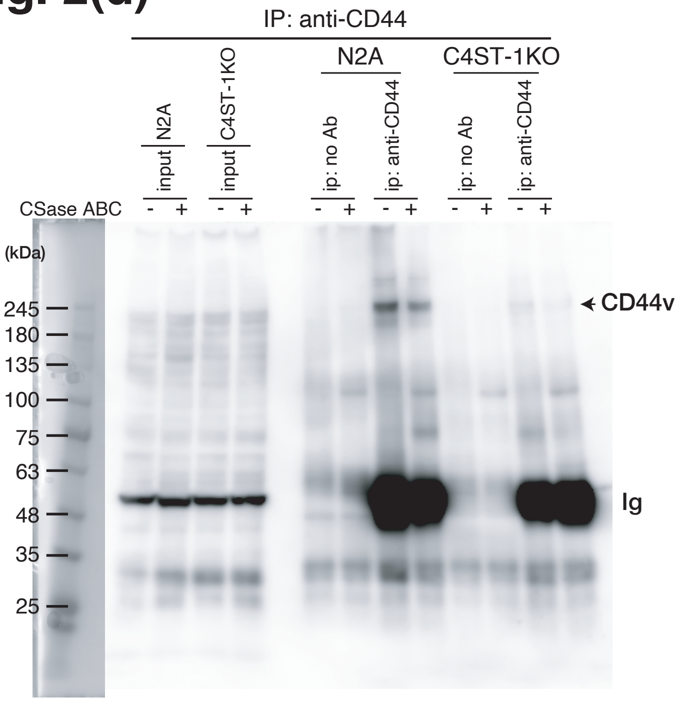

Fig. 2(e)

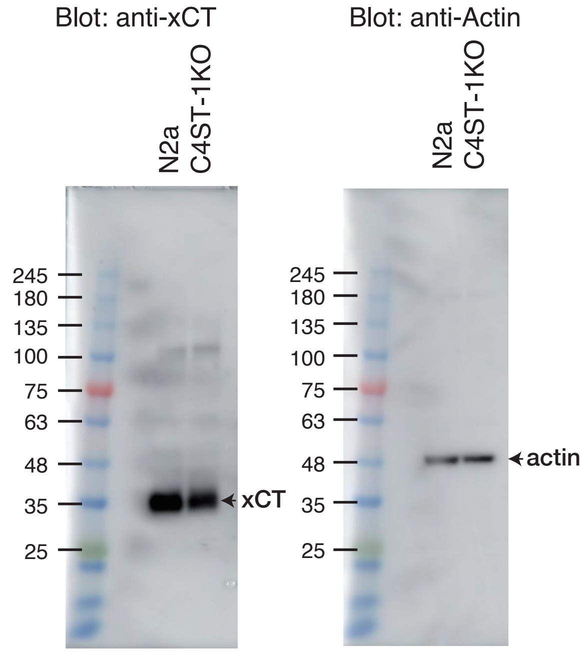

Fig. 2(e)

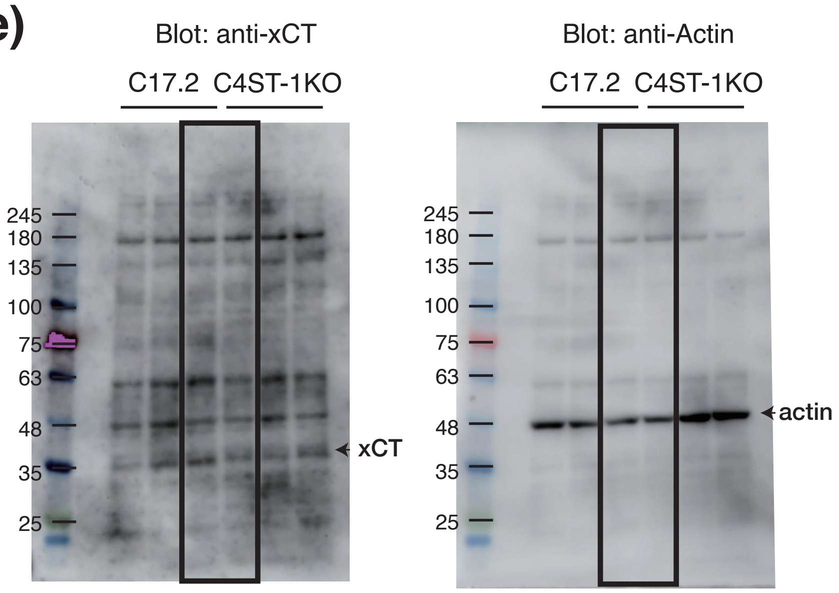

Supplementary Figure S1 (continued)

Fig. 4(a)

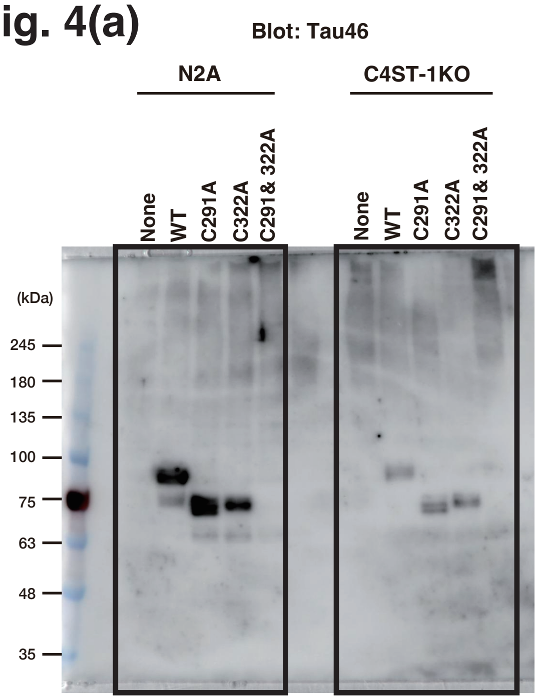

Fig. 4(c)

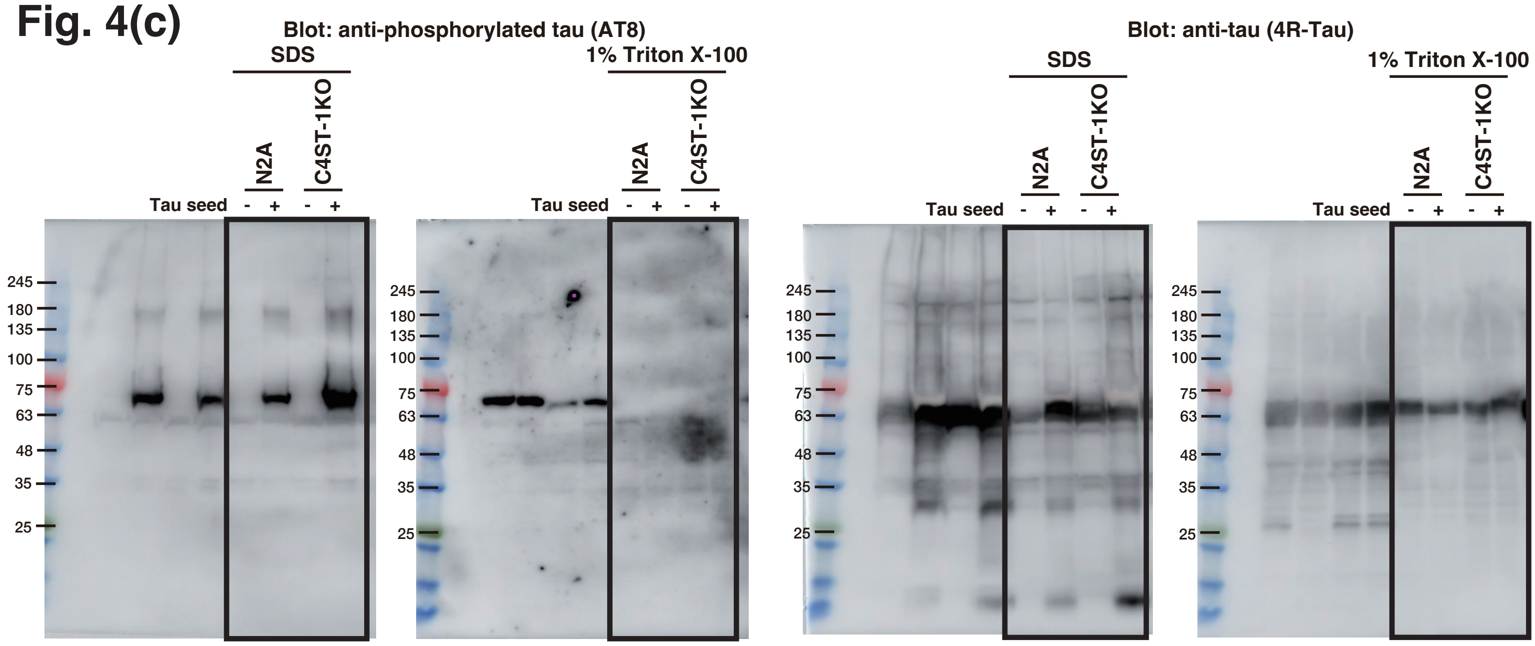

Fig. 4(d)

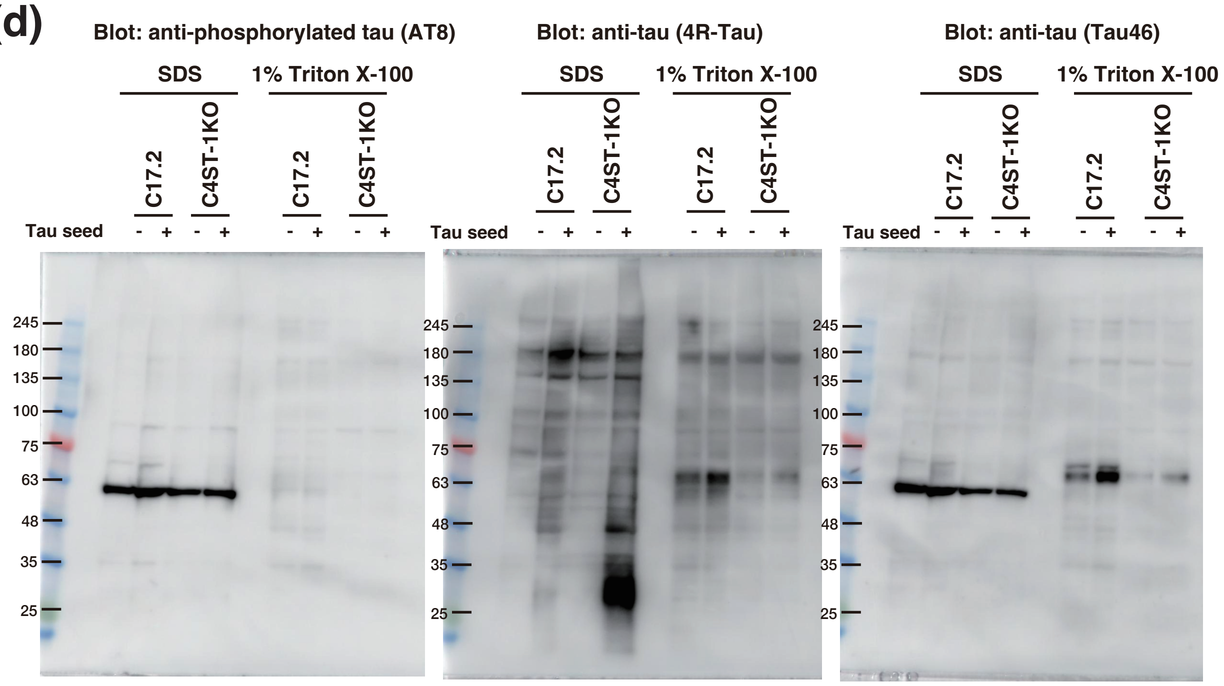

Supplement: Supplementary file 1 [file cells-14-01686-s001.zip › cells-3930909-Figure S1.pdf]
