# Supplementary material for: Dysfunctional Chondroitin 4-O-Sulfotransferase-1 Impairs Cellular Redox State and Promotes Tau Aggregation
Source: Cells. 2025 Oct 28;14(21):1686. doi: 10.3390/cells14211686 (PMC12607407; doi:10.3390/cells14211686)
Supplement: Supplementary file 1 [file cells-14-01686-s001.zip › cells-3930909-supplementary.pdf]

Supplementary Table S1

Two single-stranded oligonucleotides used to construct CRISPR nuclease vectors to knockout mouse *C4st-1* gene and sequencing primers to confirm gene editing of *C4st-1*.

	Sequence
Top oligonucleotide	5′-GCGGAGGAACCCCTTCGGTGGTTTT-3′
Bottom oligonucleotide	5′-CACCGAAGGGGTTCCTCCGCCGGTG-3′
Forward sequence primer for *C4st-1*	5 ′-TTTCTTCCTCCCTCCCAGAT-3′
Reverse sequence primer for *C4st-1*	5 ′-CAGGAAAGGGCATGTCAAAT-3′

The underlined sequences were complementary to the overhang sequence in the linearized CRISPR Nuclease Vector (Thermo Fisher Scientific).

Supplementary Table S2

Knockdown siRNA and TaqMan probes for real-time PCR used to confirm gene expression.

Gene Name	DsiRNA Sense Strand Sequence (Integrated DNA Technologies)	TaqMan Assay ID (Thermo Fisher Scientific)
mNcan#1	5′-AAGGUUUGGCUAAAGUCACUGUATC-3′	Mm00484007_m
mNcan#2	5′-CUGAUGCAGAUUCUAUAGAAAUCGA-3′	
mSdc3#1	5′-GCGUGGUGAAUGCAUGUAAAUACTT-3′	Mm01179833_m
mSdc3#2	5′-GCUACACCUUGGAAGAACCCAAGCA -3′	
mGapdh		Mm99999915_g1

The house keeping gene, *Gapdh*, was used as an internal control for quantification.

For the siControl, NC-1 (#51-01-14-03, Integrated DNA Technologies) was used.

Supplementary Table S3

Information on cell lines, reagents, and antibodies used in this study.

Cell Line Name	Company Name (Part No.)	Origin
Neuro-2a	Japanese Collection of Research Bioresources (IFO50081)	Mouse neuroblastoma
C17.2	European Collection of Cell Cultures (07062902)	A neural progenitor line derived from the mouse cerebellum

Reagent Name	Company Name Part No.	Usage
Eagle's minimal essential medium	Fujifilm Wako Chemical Corporation 056-08385	Cell culture
Non-essential amino acids	Proteintech 15675-1-AP	Cell culture
Fetal bovine serum	Sigma-aldrich 172012-500ML	Cell culture
100 units/mL penicillin<br>100 mg/mL streptomycin	Nacalai tesque 26253-84	Cell culture<br>Cell culture
GlutaMAX™ (100x)	Gibco 35050-061	Cell culture
Dulbecco's modified Eagle medium	Nacalai tesque 08459-64	Cell culture
Heat-inactivated horse serum	Thermo Fisher Scientific 26050088	Cell culture
GeneArt® CRISPR Nuclease Vector with orange fluorescent protein reporter kit	Thermo Fisher Scientific A21174	Genome editing
Lipofectamine® CRISPEMAX	Thermo Fisher Scientific CMAX00001	Transfection
Lipofectamine® RNAiMAX	Thermo Fisher Scientific 13778-075	Transfection
THUNDERBIRD® Probe qPCR Mix	Toyobo QPS-101	Real-time PCR
ROS assay kit -Highly Sensitive DCFH-DA-	Dojindo Laboratories R252	Detection of reactive oxygen species
ProteoStat® Aggresome detection kit	ENZO Life Sciences ENZ-51035	Detection of protein aggregates
Bafilomycin A1	Cell Signaling Technology 54645	Lysosomal inhibitor
MTT cell count kit	Nacalai tesque 23506-80	Measurement of live cell count
Protein-Shifter Plus	Dojindo Laboratories SB12	Labeling of free SH groups in proteins
Tau aggregation assay kit	Cosmo Bio TAU01	Detection of tau aggregation
Lipofectamine® 3000	Thermo Fisher Scientific L3000008	Transfection

Antibody Name	Company Name Part No.	Dilution	Usage

anti-xCT antibody	Abcam<br>AB307601	1:200	immunoprecipitation
anti-CD44 antibody	Proteintech<br>15675-1-AP	1:400	immunoprecipitation
anti-xCT antibody	Abcam<br>AB307601	1:8,000	Immunoblotting
anti-CS antibody<br>Clone 1-B-5	Seikagaku Corporation<br>(The produst is not available for purchase)	1:10,000	Immunoblotting
anti-CD44 antibody	Proteintech<br>15675-1-AP	1:2,000	Immunoblotting
anti-neurocan peptides antibody	Cosmo Bio Co., LTD.<br>NU-07-005	1: 5,000	Immunoblotting
Anti-Tau<br>Clone Tau46	Cell Signaling Technology<br>4019	1: 8,000	Immunoblotting
Anti-Tau<br>Clone 4R-Tau	Cosmo Bio Co., LTD.<br>TIP-4RT-P01	1:10,000	Immunoblotting
Anti-phosphorylated Tau<br>Clone AT8	Fujirebio<br>90206	1:10,000	Immunoblotting

All antibodies used for Immunoblotting were diluted in Signal Enhancer HIKARI (02270-8, Nacalai tesque).
